# An Overview of Therapeutic Options in Relapsing-remitting Multiple Sclerosis

**DOI:** 10.7759/cureus.5246

**Published:** 2019-07-26

**Authors:** Sidra Saleem, Arsalan Anwar, Muniba Fayyaz, Fatima Anwer, Faria Anwar

**Affiliations:** 1 Neurology, University of Toledo, Toledo, USA; 2 Neurology, University Hospitals Cleveland Medical Center, Cleveland, USA; 3 Internal Medicine, Fatima Memorial Hospital, Lahore, PAK; 4 Family Medicine, King Edward Medical College, Lahore, PAK; 5 Internal Medicine, Shifa International Medical College, Islamabad, PAK

**Keywords:** relapsing remitting multiple sclerosis, demyelination, autoimmune, therapeutics, deep brain lesions

## Abstract

Multiple sclerosis (MS) is a chronic disorder of the central nervous system (CNS). MS affects 2.1 million individuals every year and is also considered a major cause of economic health burden around the world. Genetics and environmental factors both play a role in the pathogenesis of MS by activating the immune response and causing inflammation. Patients with MS can have various clinical courses, but the most common pattern seen is relapsing-remitting multiple sclerosis (RRMS). Multiple therapeutic options have been studied to prevent RRMS patients from frequent relapses. The oldest and most frequently used medication for MS is interferon beta, either used alone or as add-on therapy with other drugs. Newer treatment options that have been recently approved to control MS symptoms and suppress the inflammation are glatiramer acetate and siponimod. Infusion therapies consisting of monoclonal antibodies and immunosuppressive drugs have also been studied in the recent past. Some trials have been conducted on the use of stem cells for RRMS patients. We have briefly discussed all treatment options and the response of RRMS patients in multiple trials.

## Introduction and background

Multiple sclerosis (MS) is a chronic central nervous system (CNS) disorder with widespread primary demyelination, inflammation, and progressive neurodegeneration [1]. The prevalence of MS has been rising since the last decade, affecting about 400,000 individuals every year in the U.S. and 2.1 million individuals around the world, with a male to female ratio of 2.5:1. The direct and indirect costs of MS on US healthcare ranged from $8,528 to $52,244 per patient per year [2].

The exact etiology and pathogenesis of MS are uncertain, but both genetic and environmental factors contribute to disease development. Genetic factors are associated with the activation of the immune system, involving both an innate and an adaptive pathway while environmental factors include viral infections such as Epstein Barr virus, vitamin deficiency, and smoking. The commonly suggested hypothesis is that infection within CNS or neural disturbance initiates the development of the disease, leading to immune cell activation and disease progression [3]. This autoimmune etiology is the target of the therapeutic approach to MS patients, but many patients still face relapses and continuous deterioration, progression, brain atrophy, and new magnetic resonance imaging (MRI) lesions. Our objective is to describe the comprehensive overview of different therapy options for these relapse patients and to give the reader a better understanding of the treatment approach in these patients.

## Review

Types of multiple sclerosis

Patients with MS may have a different clinical course. In most patients, the disease presents as the relapsing-remitting course (RRMS). It is the most prevalent form, affecting 85% of individuals. RRMS is marked by discrete, day-to-week attacks, followed by a week-to-month symptom-free interval. RRMS can progress to secondary progressive multiple sclerosis (SPMS) that is characterized by an initial relapse, followed by gradual neurologic deterioration. Many patients can present with primary progressive disease (PPMS) with a slow decline from the start. In a very few patients, the disease manifests as a continuous decline in function from the onset and later by an acute attack defined as progressive relapsing multiple sclerosis (PRMS) [4].

Treatment of relapsing-remitting multiple sclerosis

The National MS Society has identified more than 136 studies to evaluate the different therapeutic options for MS. Food and Drug Administration (FDA) has approved the following drugs for RRMS, which will be discussed in detail [5].

1) Injectables (Interferon and Glatiramer Acetate)

2) Oral Therapies (Fingolimod, Teriflunomide, Dimethyl fumarate, Siponimod, Cladribine)

3) Infusion Therapies (Natalizumab, Ocrelizumab, Alemtuzumab, and Rituximab)

4) Other (Azathioprine, Laquinimod, Cyclophosphamide, Daclizumab, Dalfampridine, Mitoxantrone, Glucocorticoids, Intravenous immunoglobulin (IVIG), Dalfampridine)

Injectables

Interferon-Beta

This drug was approved for MS therapy by the FDA in 1993. Its mechanism of action is multifold and includes the inhibition of T-cell activation and a decrease in the activity of matrix metalloproteinase. It decreases proinflammatory cytokines and leads to the production of anti-inflammatory cytokines by increasing the activity of suppressor T-cells [6].

Route of administration: Subcutaneous/intramuscular

The literature search and original articles for the treatment of interferon-beta in RRMS are summarized below (Table 1).

**Table 1:** Clinical trials for the use of interferon-beta in RRMS RRMS: relapsing-remitting course

Study, country	Sample size, duration	Methodology	Results
Calabresi et al. [7], 2014, involved 26 countries.	1516 patients, 2 years.	It was a double-blind, phase III, placebo-controlled design for 48 weeks. Patients were randomly assigned as 500 to Placebo, 512 to Pegylated Interferon (PEG-INF) every 2 weeks group and 500 patients to PEG-INF every 4 weeks group. The primary endpoint at 48 weeks was measured.	After 48 weeks, PEG-INF group had fewer relapses than the placebo group.
Moccia et al. [8], 2017, Italy.	507 patients, 8.5 ± 3.9 years.	Subjects were divided into 3 groups, 37% were given subcutaneous Interferon-β1a 44mcg, 33.4% with intramuscular Interferon-β1a 30mcg, and 29.0% with subcutaneous Interferon-β1b 250mcg.	Formulation, frequency, and dosage of Interferon-β1a can affect long-term clinical efficacy as a 44 mcg group showed more reduction in disability as compared to two other groups.
Drulovic et al. [9], 2013, Europe.	419 patients,7 years.	Subjects were divided into two groups, 236 IFN-beta-treated, and 183 control. They were followed for 7 years and the number of relapses was noted.	The group treated with interferon showed a significant reduction in progression to secondary progressive MS.
Saida et al. [10], 2016, Japan.	100 patients, 2 years.	All subjects were given intramuscular IFN beta-1a, one was given intramuscular 30mcg/weekly and other was given 15mcg/week for 2 weeks and then 30mcg/week.	Intramuscular Interferon has reduced the relapsing rate with a good safety profile.
Traboulsee et al. [11], 2018, Canada	560 patients, 1 year.	Subjects were divided into 3 groups, One group with IFN β-1a subcutaneously 44 μg, second group with IFN β-1a subcutaneously 22 μg three times weekly and the third group as placebo-controlled. All were followed for a year and clinical and radiological improvement was compared.	Clinical and radiological improvement with no evidence of disease activity was noted in Interferon group as compared to placebo.

Glatiramer Acetate

Glatiramer acetate (GA) was approved for the treatment of RRMS by the FDA in 2018. Its mechanism of action is two-fold, immunomodulatory and neuroprotective. Its immunomodulatory action is due to its competition by binding to antigen-presenting cells, driving B-cells, monocytes, and dendritic cells towards an anti-inflammatory response, induction of T-regulatory/Th2/Th3 cells, and down-regulating Th17/Th1 cells. Its neuroprotective effect is due to the secretion of neurotrophic factors that help in remyelination [12].

Route of administration: Subcutaneous

The literature search and original articles for the use of GA in RRMS are summarized below (Table 2).

**Table 2:** Clinical trials for the use of glatiramer acetate (GA) in RRMS RRMS: relapsing-remitting course

Year, Study	Sample size, Duration	Methodology	Results
Rio et al. [13], 2014, Spain.	151 patients, 1 year.	GA was given to patients with RRMS and followed for one year. Clinical and radiological activities were noted.	Patients treated with GA has improved clinical and radiological findings.
Wolinsky et al. [14], 2015, 31 states of the US.	209 patients, 4 months.	Subjects were randomized in 1:1 ratio to GA 20 mg/ml and GA 40 mg/ml group and were followed for 4 months and relapses were noted.	The response rate was better in patients taking 40 mg GA.
Ziemssen et al. [15], 2014, 148 centers worldwide.	672 patients, 24 months.	All RRMS patients that were on any kind of disease-modifying therapy were included. Their therapy was converted to 20 mg/ml GA. Patients were assessed at baseline and at 6,12,18 and 24 months.	Results showed that with the conversion to GA, patients showed improvement in cognition, fatigue, and quality of life and their disability scale was stable.
Davis et al. [16], 2017, 17 countries.	1404 patients, 1 year.	Patients with RRMS was given GA 40mg/ml mg and followed clinically and radiologically.	GA decreased 30% of relapse within 2 months of therapy
Zipser et al. [17], 2015, Australia	19 patients, 24 months	12 patients with RRMS were started on GA and 7 patients as control were included. All patients were followed for 24 months.	The results of this study contradict the neuroprotective effect of GA and do not show an improvement in paraclinical markers.

Oral therapies

The following five oral drugs have been approved by the FDA for the treatment of RRMS.

Fingolimod

Fingolimod was first approved by the FDA in 2010. It is an analog of sphingosine and thereby modulates the sphingosine-1-phosphate receptor that alters the migration and sequestration of lymphocytes in the lymph nodes [18].

Route of administration: Oral

The literature search and original articles for the use of fingolimod in RRMS are summarized below (Table 3).

**Table 3:** Clinical trials for the use of fingolimod in RRMS RRMS: relapsing-remitting course

Year, Study	Sample Size, Duration	Methodology	Results
Kappos et al. [19], 2015, Switzerland.	1272 patients, 2 years.	All RRMS patients received 0.5, 1.25mg of Fingolimod daily or placebo. The primary endpoint was annualized relapse rate and the secondary endpoint was time to disability progression.	Relapse rate, the risk of disability progression and end points on MRI were improved with both doses (0.5 and 1.25mg) as compared to Placebo.
Saida et al. [20], 2012, Japan.	171 patients, 6 months.	All patients with RRMS either received 0.5mg or 1.25mg of Fingolimod or placebo daily. The primary endpoint was the percentage of patients with a Gadolinium-enhanced lesion at 3 and 6 months and the secondary endpoint was relapsed at 6 months.	Relapse rate and Gadolinium lesions were improved in Fingolimod group as compared to placebo.
Kappos et al. [21], 2006, Switzerland.	281 patients, 6 months.	All patients with MS are given fingolimod at a dose of 1.25 or 5.0mg or placebo. Primary Endpoint was the total number of Gadolinium-enhanced MRI at monthly intervals for 6 months and clinical evaluations.	After 6 months, Fingolimod reduced the number of lesions on MRI and improved clinical outcome at both doses as compared to Placebo.
Cohen et al. [22], 2010, USA.	1292 patients, 1 year.	Patients were given either 1.25mg or 0.5mg of Fingolimod daily or 30 mcg of intramuscular interferon beta-1a. The primary endpoint was annualized relapse rate and the secondary endpoint was a new lesion in MRI after 12 months.	The trial showed Fingolimod improved outcomes with respect to relapse rate and MRI findings, in comparison with Interferon beta-1a.

Dimethyl Fumarate

Dimethyl fumarate (DMF) was approved by the FDA in March 2013. Its complete mechanism of action is not fully understood yet, although its neuroprotective and anti-inflammatory properties are due to the activation of Nrf2 pathways [23]. Multiple studies were conducted to show the effectiveness of DMF. Fox et al. [24] conducted a study by including 1400 patients with RRMS and were given DMF 480 mg, 720 mg, and subcutaneous GA 20 mg or placebo in a ratio of 1:1:1:1. Patients were followed for two years and the number of relapses was noted. After the completion of the study, both doses of DMF and GA proved to be more effective in reducing the relapse rate and improving neuroradiologic findings [24]. In 2010, another study in Germany showed the same result [25]. DMF treatment may decrease the lymphocyte count, and if lymphopenia develops, it is recommended to stop DMF. Another concern is liver injury, which can lead to increased aminotransferase [26].

Teriflunomide

This drug was approved by the FDA in September 2012. It works as an immunomodulator and is the active leflunomide metabolite that inhibits the biosynthesis of pyrimidine and disrupts the interaction between T-cells and antigen-presenting cells (APCs) [27]. Confavreux et al. [28] conducted a study in which 1088 patients were assessed for the effectiveness of teriflunomide. The annual relapse rate and disability improvement were noticed. After two years, the results showed a significant reduction in relapse rate, disability progression, and disease activity on MRI. Another study was conducted in France [29], in which they assessed the efficacy of teriflunomide in patients recruited from 189 sites in 26 countries and randomly assigned into the placebo group, teriflunomide 7 mg, and 14 mg in a ratio of 1:1:1. Primary endpoints were relapse rate and disability progression. Results showed a better efficacy of 14 mg in preventing relapse and improving disability. Teriflunomide should not be provided due to the danger of hepatotoxicity to patients with chronic liver diseases and is also contraindicated due to teratogenicity in pregnant females [30].

Siponimod

Siponimod is similar to fingolimod but more selective, and it was approved by the FDA in March 2019 for adults with RRMS, SPMS, and clinically isolated syndromes [31]. Several studies were conducted to determine the effectiveness of siponimod in RRMS and these showed that it can reduce the frequency of relapse and brain lesions on MRI [32]. Siponimod is contraindicated in patients with CYP2CP9*3/*3 or those with advanced heart failure, myocardial infarction, stroke, or transient ischemic attack (TIA) [33].

Cladribine

It was first approved by the FDA in March 2019 for the treatment of RRMS and SPMS. It is a purine antimetabolite, hence acting as an immunosuppressive agent and decreasing the lymphocyte count [34]. Giovannoni et al. [35] in their trial included 1326 adults with RRMS in a randomized double-blind study showed the efficacy of cladribine. Patients were given 3.5 mg/kg or 5.25 mg/kg of cladribine or placebo. Primary endpoints assessed at 96 weeks showed a decrease in relapse rate, disability progression, and MRI lesions. Mild to moderate lymphocytopenia was also seen with a high and low dose of cladribine.

Infusion therapies

Infusion therapies comprise a group of medications that acts against B-cells to suppress the autoimmune response. Several drugs have been tested for treatment in MS, including natalizumab, alemtuzumab, ocrelizumab, and rituximab. Natalizumab was approved by the FDA in November 2005 and is directed against integrin molecules, resulting in decreased adhesion and the migration of leukocytes. Alemtuzumab was approved by the FDA in November 2014, and it is a humanized monoclonal antibody that causes the depletion of CD52-expressing T-cells, B-cells, natural killer cells, and monocytes [24]. Ocrelizumab was approved by the FDA in March 2017 and is a recombinant human anti-CD20 (a B-cell marker) monoclonal antibody. It optimizes B-cell depletion by the modification of the Fc region, which enhances antibody-dependent cell-mediated cytotoxicity and reduces complement-dependent cytotoxicity. Rituximab was approved by the FDA for multiple disorders, but it is also used by neurologists as off-label, and it has a similar mechanism of action as ocrelizumab [36]. The literature search and original articles for the use of infusion therapies in RRMS are summarized below (Table 4).

**Table 4:** Clinical trials summarize the use of infusion therapies in RRMS RRMS: relapsing-remitting course

Year, Study	Sample size, Duration	Methodology	Results
Polman et al. [37], 2016, Netherland.	942 patients, 2 years.	All patients receive natalizumab 300 mg or placebo. Rate of relapse and progression of disability were measured.	Natalizumab reduced the disability progression and clinical rate of relapse and proved to be an effective treatment for RRMS.
Rudick et al. [38], 2006, USA.	1171 patients, 2 years.	All patients with RRMS were assigned to receive natalizumab added to interferon beta 1a or placebo in a double-blind study. Primary endpoints were clinical relapse at 1 year and disability progression assessment at 2 years.	Natalizumab added to Interferon beta 1a proved to be more effective than interferon beta 1a alone in reducing clinical relapse and also lowered the disability progression.
Cohen at al. [39], 2011, Switzerland.	581 patients, 2 years.	Previously untreated RRMS were included and allocated into 2:1 ratio to receive Alemtuzumab 12mg per day or subcutaneous interferon beta 1a 44μg. Primary endpoints were relapse rate and disability assessment for 2 years at 6 months interval.	Results showed that with the addition of Alemtuzumab, there was a 19% improvement in clinical relapse and disability than interferon beta 1a alone.
Coles et al. [40], 2012, UK.	638 patients, 2 years.	All RRMS patients were given interferon beta 1a, Alemtuzumab 12 mg per day or alemtuzumab 24 mg per day in a ratio of 1:2:2. Relapse rate and evaluation of disability were done every 6 months for 2 years.	In patients with RRMS, Alemtuzumab can be used to reduce the relapse rate and time and improve disability in patients.
Hauser et al. [41], 2017, USA.	1656 patients, 22 months.	Patients were given Ocrelizumab 600 mg every 24 weeks or subcutaneous Interferon beta 1a at a dose of 44ug three times per week for 96 weeks. The number of relapses was noted.	Patients with Ocrelizumab showed fewer relapses as compared to patients receiving interferon beta 1a.
Kappos et al. [42], 2011, 20 countries	220 patients, 4 months	Patients were divided into 3 groups, placebo, low-dose (600 mg) or high-dose (2000 mg) ocrelizumab and intramuscular interferon beta-1a (30μg). Primary endpoints were Gadolinium-enhanced lesion (GEL) and MRI at multiple week intervals.	Depletion of B-cells with both Ocrelizumab doses was seen and it also supports the role of B-cells in the pathogenesis.
Hauser et al. [43], 2008, USA	104 patients, 4 months	All RRMS patients were randomized to receive 1000 mg of Rituximab and placebo. The primary endpoint was the total count of GEL on MRI at multiple weeks interval.	Rituximab reduced inflammation and lesion on MRI.

Others

Several immunosuppressants and immunomodulators have been evaluated for RRMS treatment, including mitoxantrone, cyclophosphamide, azathioprine, methotrexate, and mycophenolate mofetil. However, only the first three have shown better efficacy in the treatment of RRMS and are described below.

Mitoxantrone

Mitoxantrone was approved by the FDA in 2000 for treatment of RRMS. Its mechanism of action is manyfold; it inhibits topoisomerase II, intercalates deoxyribonucleic acid (DNA), and suppresses immune cells and secretions of cytokines. Its efficacy in the treatment of RRMS is questionable due to its increased adverse effects [44]. Thus, it is only considered as a last resort in patients with a rapidly progressing disease where other medications have failed.

Cyclophosphamide

This drug is unique in its blood-brain barrier-crossing property. Its mechanism of action is due to immunosuppression by shifting the Th1 to a Th2 response and breaking the DNA. It has a good early-stage effect in which inflammation is superseded by degeneration. It is usually used as pulse therapy in patients who are not responding to INFs or GA [45]. In a study by Krishnan et al., patients with RRMS were treated at a dose of 50 mg/kg/day with IV cyclophosphamide for four days and then a leukocyte colony-stimulating factor was given. After 23 months, patients were evaluated and significant disability reduction was noted [46] but more studies are needed to determine its effectiveness.

Azathioprine

It is used as an alternative to interferon (INF) in some patients of RRMS. It is a purine analog that inhibits DNA synthesis and suppresses immunity. Casetta et al. [47] in their systematic review included five randomized control trials with 698 patients. The azathioprine group showed a significant reduction in relapses of MS patients as compared to placebo when observed at the first, second, and third years of treatment. In one trial, it was found that if azathioprine is given at a dose of 3 mg/kg/day, it can significantly reduce lesions in MRI [48].

Autologous stem cell transplantation

Stem cells have been well-investigated for the treatment of RRMS. The basic concept behind this therapy is harvesting the patient’s own blood cells, treating these cells with chemotherapy and biologics to destroy autoimmune cells, and the reinfusion of these stem cells into patients to regenerate the immune system. Several studies have been conducted to explain the role of stem cells in the treatment of MS Burt et al. [49] in their study included 110 patients with two relapses while being treated with conventional treatment. These patients were observed for a year and the Expanded Disability Status Scale (EDSS) was measured at six months and one year. They concluded that treatment with stem cells results in a prolonged time to disease progression. In their study, Nash et al. [50] included 24 patients with refractory RRMS. These patients were given high-dose immunosuppression and resulted in a three and five event-free survival year (78% and 69%, respectively) and improvements in neurologic function [50]. However, to illustrate the cost-benefit result, these trials are limited and more data is required to make it conventional.

## Conclusions

The treatment of frequent relapses in patients with MS is a common challenge for neurologists. Many studies have been conducted to evaluate treatment options that can decrease the frequency of relapses and improvement in radiological lesions. All therapeutic options have been focussed on slowing neurodegeneration and decreasing inflammation. However, to give promising options to RRMS patients, we still need standard guidelines for dosage, frequency, and add-on treatment options.
